# Observed Aspects of Mate Value and Sociosexuality Account for Mate Preferences: Data from a Large, Representative Study from Czechia

**DOI:** 10.1007/s10508-024-03010-4

**Published:** 2024-10-21

**Authors:** Zsófia Csajbók, Zuzana Štěrbová, Peter K. Jonason, Lucie Jelínková, Jakub Binter, Jan Havlíček

**Affiliations:** 1 Faculty of Humanities, Charles University, Prague, Czech Republic; 2 Faculty of Science, Charles University, Prague, Czech Republic; 3 Faculty of Arts, Charles University, Prague, Czech Republic; 4 Psychology Research Institute, University of Economics and Human Sciences, Warsaw, Poland; 5 Faculty of Social and Economic Studies, University of Jan Evangelista Purkyně, Ústí nad Labem, Czech Republic

**Keywords:** Mate preferences, Mating strategy, Mate value, Sex differences, Desirability, Undesirability

## Abstract

While there is substantial research on what people want in their romantic and sexual partners, much of this work focuses on WEIRD, youthful samples, fails to consider the role of undesirable characteristics (i.e., things people do not want in partners) at all, or in conjunction with desirable characteristics (i.e., things people do want in partners), and may be overly reliant on psychometric approaches to pivotal variables in mating psychology like mate value and sociosexuality. In a nationally representative (online) sample of 2280 people from Czechia (aged between 18 and 50 years old), we examined linear and quadratic age, education, and self-perceived mate value (desirability) effects on the desired levels in mate choice of eight undesirable and seven desirable characteristics in men and women in relation to ostensible metrics of mate value. Self-perceived mate value alone explained little variance (men 1%, women 2%), while all mate value and mating strategy indicators together explained little variance of mate preferences and aversions (men 3%, women 5%). Desirable characteristics were better explained by mate value than undesirable ones. Our results are in line with evolutionary predictions suggesting that women are more demanding. Also, more qualities to offer correlate with more expectations in a partner.

**Supplementary Information:**

The online version contains supplementary material available at 10.1007/s10508-024-03010-4.

## Introduction

What people want and do not want in their partners is, in theory, idiosyncratic. Several studies have simply relied on a list of researcher-chosen characteristics that people might like or not in their partners (e.g., Buss, 1989; Furnham, 2009). They found systematic, cross-nationally similar (but not identical) mate preferences (Jonason & Thomas, 2022; Thomas et al., 2020; Walter et al., 2020). Most research has focused on preferences, although undesirable characteristics (i.e., aversions; characteristics that prevent the formation or maintenance of a relationship) are pivotal to a holistic view of mate choice (Jonason et al., 2015).

Evolutionary-informed researchers propose that the ultimate function of having a mental image of avoidable characteristics is to avoid mating and health risks, including physical costs, pathogen exposure, and psychological problems (Carton & Egan, 2017; Little et al., 2011). As such, when potential partners show cues of low quality, people should rate those potential partners as less desirable. Dating an unambitious, filthy, hostile, clingy, or depressed partner will and has likely imposed costs on people who are indiscriminate in their mate choice (Hammen & Peters, 1978). For instance, being “filthy” may serve as a warning signal of risk for infection and, indeed, disgust systems may have evolved to protect people from the recurrent risks posed by the uniquely ultrasocial way of human life (Al-Shawaf et al., 2019). Further, dating someone abusive, depressed, hostile, or arrogant may impose relationship costs making the relationship harder to maintain. Investigating aversions is also fruitful from the point of view that, unlike ideal preferences, people may be more aware of dealbreakers, that is, undesirable characteristics (Jonason et al., 2015; e.g., my partner’s ideal body height is 1.8m, but he must not be shorter than me).

### Sex and Age Differences in Mate Preferences and Aversions

The most well-investigated variable to account for variance in mate preferences is whether someone is a man or a woman which is unsurprising given the recurrent asymmetries in reproductive investment in the sexes (Trivers, 1972). One of the most fundamental concerns for humans is that their offspring are helpless for an extended period which requires substantial parental investment. While modern welfare systems may protect single mothers today, the need for a warm, resourceful, and good partner in parenting originates from ancestral pressures (Trivers, 1972). Because the minimum necessary investment in reproduction is higher for women (and female mammals), we would expect they will want a partner who is warm and has high resource acquisition potential such as high status and dominance more than how important it is for men (Jonason & Thomas, 2022; Jonason et al., 2012). In contrast, while both sexes want a partner who is physically attractive (Li, 2007), men may prioritize this trait more than women because it may serve as a cue to fecundity and fertility (Singh, 2002).

Sex differences in mate preferences have been investigated extensively during the last decades; however, these studies focused mainly on university students and young adults between 18 and 35 years of age (Buss, 1989; Evans & Brase, 2007; Moore et al., 2010). The age-related shift in mate preferences includes decreased preferences in physical characteristics (e.g., curvaceous) but increased preferences for softheartedness and conventionality (Brumbaugh & Wood, 2013); a change that was stronger in women. Further, people in their 30’s do not put as strong emphasis on physical attractiveness as people in their 20’s; however, men’s preferences for physical appearance decreased more steeply than women’s as they were growing older (Sprecher et al., 2019; but see Apostolou & Eleftheriou, 2022). On the other hand, women were more interested in social status and preferred men with a stable income, but this preference increased only until their 40s, then it declined (Fales et al., 2016). In terms of age preferences, men showed relatively stable preferences for women younger than themselves throughout their lives (Kenrick & Keefe, 1992; Kenrick et al., 1996), although the tolerated age gap between the partners increases with age in men, but it is relatively stable in women (Schwarz & Hassebrauck, 2012).

Age also correlates with preferences that are not as clearly sex-specific as status and physical attractiveness. While growing older, people are looking for a more educated partner. This tendency was more pronounced in men, who preferred more intelligent partners, while women preferred a more dominant partner while aging (Buunk et al., 2002). In older age, both men and women preferred more creative and domestic partners (Schwarz & Hassebrauck, 2012). Lastly, online dating studies suggested that while men became more demanding in their preferences with age, women lowered their expectations (De Sousa Campos et al., 2002).^1^

In contrast to sex differences in mate preferences, there is less work on sex differences in mate aversions, but the evidence does align with parental investment theory (Trivers, 1972). From this perspective, the sex that pays heavier costs for mating mistakes will have stronger and more sensitive rejection systems. For instance, when men and women learn an additional favorable characteristic about a potential partner, they both go up in a similar amount of interest, but when learning about something undesirable, women discount (i.e., lose interest in) that partner more heavily than men do (Jonason et al., 2015). This may mean that larger sex differences may emerge for mate aversions as women need to protect their interests more than men. Take depression as an example. In families and romantic couples, more collateral damage is created when the man is depressed than when the woman is (Hammen & Peters, 1977, 1978). Along the same line, a longitudinal study revealed a group of couples with only women having constantly high depressive symptoms while men having constantly low depressive symptoms but not vice versa (Csajbók et al., 2022).

### Individual Differences in Mate Preferences and Aversions

Two of the most central individual differences in predicting mate preferences/aversions are mating strategies (Li, 2007) and mate value (Regan, 1998). Mating strategies (long-term and short-term) are usually captured with questions about people’s interest in romantic/sexual relationships (Buss & Schmitt, 1993, 2019) or individual differences in how favorable someone feels about casual sex (Schmitt, 2005). Although sexual strategies theory has faced criticism (such as the oversimplification of human behavior into short-term and long-term strategies or the neglection of cultural, social, and individual variation; Fletcher et al., 2015; Schmitt, 2007), it remains an important approach to understanding human mating behavior. Beyond psychometric scales (Edlund & Sagarin, 2014), we might capture these individual differences by asking people about how many children they have had (Pflüger et al., 2012), how many sex partners they have had (Jonason et al., 2009), and how many romantic relationships they have had (Fisher et al., 2008).

Alternatively, mate value is the degree to which someone embodies qualities that make them desirable as a romantic/sexual partner (e.g., Kirsner et al., 2003). As correlates of mate value one can test alternative measures like participant's age and it’s square (Csajbók et al., 2023), income, or level of education (Jonason & Thomas, 2022). Age and its square predict mate value differently in men and women. People with more income and education (at least in Western cultures) are viewed as more desirable as romantic/sexual partners. In the current study, instead of serving as indicators of internal mating systems, by focusing on life outcome data as opposed to psychometric scales, we correlate mate preferences/aversions with real-life outcomes and self-perception, not hypothesized psychological systems.

Individual differences in mate value also predict preferences because of assortative mating. People are more likely to be similar to their partner than dissimilar and homogamy was observed along multiple characteristics and in preferences for self-similarity as well (Luo, 2017; Watson et al., 2014). Therefore, those with higher mate value should be more discriminating, rating people with less desirable and more undesirable characteristics less favorably than those with lower mate value (Csajbók & Berkics, 2022; Jonason et al., 2015). That is, mate value operates as “buying power” in a “mating market” where greater power translates to a stronger ability to exercise one’s preferred mating strategy (Conroy-Beam, 2018). However, we also measure self-perceived mate value because it may be a more powerful predictor of relationship preferences/aversions because one’s beliefs about oneself are likely better predictors of their preferences than their actual life outcome data (Arnocky, 2018; Edlund & Sagarin, 2014). In contrast, having more sexual and romantic relationships may translate into a tendency to reject fewer romantic/sexual partners because (1) a strong rejection system would undermine one’s success in the dating world and (2) those who are more flexible/forgiving in their mate preferences will have—in theory—more sexual and romantic partners (Csajbók & Berkics, 2022). We also explore how these correlations may be moderated by the participant’s sex.

In this study, we tested the effect of age, sex, mate value, and mating strategies on partner preferences and aversions. Uniquely, we retest prior assertions that mate value and sociosexuality are pivotal to understanding mate preferences on either side of the decision-making process (Fisher et al., 2008; Jonason et al., 2015; Regan, 1998). Here we assessed “mate value” and “sociosexuality” with a limited reliance on psychometric scales. Additionally, because of the possible nonlinear relationship between mate preferences and age (e.g., one should expect a steeper decrease in preferences related to reproduction after menopause; Brase & Guy, 2004; Csajbók et al., 2023), we further explore the way age and age-squared predict mate preferences and aversions. Lastly, we test whether so-called objective or observed aspects of mate value like level of education are useful in relation to self-perceived ones in accounting for mate preferences and aversions (Csajbók et al., 2019, 2023).

## Method

### Participants and Procedure

We sampled 2,682 participants, aged between 18 and 50, via the Czech National Panel, to take an online study for financial reward.^2^ Participants were invited to participate to meet the following quotas: sex (female, male), age (18–24yo, 25–34yo, 35–44yo, 45–50yo), income, and education (elementary, high school, university). After we excluded 277 people who did not provide complete data, and those who were reported homosexual (4.1%) and bisexual (0.8%) preferences, we were left with 2,280 people, aged between 18 and 50 (*M* = 34.57, *SD* = 9.37) who primarily were women (54.4%), in a current relationship (81.2%), and had a primary level of education (primary and secondary without a high school diploma, 34.1%).^3^^,^^4^ Participants were informed about the nature of the study and if they consented via tick-box, they proceeded through a series of self-report questions and were debriefed, thanked, and paid upon completion (around 5 EUR in Czech crowns).

### Measures

We measured three classes of variables to understand mate preferences. First, we measured several indicators of mate value. We wanted to compare measuring it with a self-reported measure of mate value that has high external and face validity (Csajbók et al., 2023) and with objective indicators (Brase & Guy, 2004). Thus, for self-perceived mate value, participants rated “How desirable do you think you are to others as a potential partner?” (1 = *very undesirable,* 7 = *very desirable*).^5^ We treated level of education (1 = *elementary or unfinished*, 5 = *university education*), personal monthly net income (1 = *less than 10, 000 CZK*, 11 = *100,000 CZK and more*), age, and age-squared as observed metrics of mate value. These metrics were uncorrelated for age and education (*r* < 0.01) and moderately correlated for income and age (*r* = 0.33; Supplementary Table S3).

Second, we assessed indicators of mating strategies with several life outcome metrics. We asked participants to report their number of children, relationships, and sex partners. Participants in a committed relationship (*n* = 1851) were asked to report its length in months. These metrics were correlated between not at all (*r* = 0.01; *N* of children with *N* of sex partners) and substantially (*r* = 0.70; *N* of partners with *N* of sex partners; Supplementary Table S4).

Next, to measure mate preferences, we assembled a list of seven desirable (i.e., Warmth, Attractiveness, Status, Intellect, Passion, Stability, and Dominance; Csajbók & Berkics, 2017) and seven undesirable (i.e., Hostility, Unattractiveness, Unambitiousness, Filthiness, Arrogance, Clinginess, and Abusiveness; Csajbók & Berkics, 2022) factors and added one additional, Depressiveness (i.e., pessimistic and depressed) because of its role in personal and relationship health (Li & Johnson, 2018; Lim et al., 2018). All 15 randomized factors were indicated by two characteristics that loaded on the factors in the original research (e.g., “loving and caring” for Warmth; “selfish and arrogant” for Arrogance). Participants were asked, “To what extent do the following characteristics describe your ideal partner?” (1 = *not at all*, 7 = *very much*).^6^ The ratings of all 15 mate preferences were correlated, but not so much to consider them redundant (i.e., *r* >|.90|; Supplementary Table S5).

Last, we captured basic demographic details. We asked participants about their city size by determining the number of residents where they lived in the 14 regions of the Czech Republic (1 = *below 1,000 residents*, 6 = *above 100,000 residents*). We also asked people how many people lived in their household and the net household (as opposed to personal like above) income. These metrics were correlated between *r* < 0.01 (size of residence with household income) and *r* = 0.28 (*N* people in the household with household income; Supplementary Table S6).

### Data Analysis

We ran 2 × 7 (desirable characteristic) and 2 × 8 (undesirable characteristic) mixed model ANOVAs to understand sex differences in mate preferences/aversions, the preferred level of each preference/aversion, and their interaction; leaning on *t-*tests and repeated-measures ANOVAs across the sexes to understand these effects. Pearson correlation coefficients were used to understand how self-perceived mate value, mate value indicators, mating strategies, sociosexuality, and demographic factors were related to mate preferences and aversions and tested whether they differed in men and women with Fisher’s *z*. Subsequently, we ran regression models to predict each ideal preference factor across men and women using the demographic variables and age-squared included. We used multiple regression to estimate the isolated and relative effects of self-perceived mate value, mate value indicators, mating strategies, sociosexuality, and demographic factors in predicting mate preferences and aversions. Ideal partner preferences along the 15 factors measured were plotted against age (having a common yet complicated assumption about its relationship with mate preferences) using the quadratic term moderated by sex in regression analysis to estimate curved associations. The same analyses were conducted and plotted against education (an objective mate value indicator with the most frequent sex moderation in the current study) and self-perceived mate value (a variable with a better expected predictive validity, Csajbók et al., 2019, 2023). The age, education, and mate value figures were created using R (1.4.1717), and the other analyses were conducted in SPSS (v26) apart from the Fisher’s *z* tests which were calculated online (psychometrica.de).

## Results

Descriptive statistics and *t*-tests comparing sex differences in demographic characteristics are reported in Table 1. Education, personal and household income, number of relationships, and size of residence were higher in men than women. In contrast, women reported having more children than men and proportionally more women were in a relationship than men.

**Table 1:** Descriptive statistics of the overall sample and sex differences

	*M* (SD)			*t/*χ^*2* a^	*d/V*
	Overall	Women	Men		
*Desirable factors*					
Warmth	6.07_a_ (1.19)	6.15_a_ (1.19)	5.98_a_ (1.20)	3.38**	−0.14
Attractive	5.50_bd_ (1.31)	5.42_b_ (1.34)	5.60_b_ (1.27)	−3.25**	0.14
Status	4.66_c_ (1.51)	4.93_c_ (1.46)	4.35_c_ (1.51)	9.34**	−0.39
Intelligent	5.55_bd_ (1.26)	5.58_d_ (1.26)	5.51_bd_ (1.26)	1.33	−0.06
Passionate	5.57_bd_ (1.39)	5.60_d_ (1.38)	5.53_b_ (1.41)	1.25	−0.05
Stable	5.43_b_ (1.49)	5.47_bd_ (1.51)	5.37_d_ (1.46)	1.65	−0.07
Dominant	5.20_e_ (1.34)	5.33_b_ (1.34)	5.05_e_ (1.33)	5.14**	−0.22
*Undesirable factors*					
Unambitious	2.20_a_ (1.47)	1.99_a_ (1.42)	2.45_a_ (1.48)	−7.57**	0.32
Hostile	1.49_b_ (1.13)	1.38_b_ (1.02)	1.62_b_ (1.24)	−5.05**	0.21
Filthy	1.82_c_ (1.43)	1.80_c_ (1.46)	1.85_c_ (1.39)	−0.81	0.03
Arrogant	1.79_c_ (1.32)	1.72_c_ (1.3)	1.88_c_ (1.34)	−2.92**	0.12
Unattractive	1.88_c_ (1.39)	1.86_ac_ (1.39)	1.91_ cd_ (1.40)	−0.81	0.03
Clingy	4.03_d_ (1.83)	3.91_d_ (1.91)	4.17_e_ (1.71)	−3.36**	0.14
Abusive	1.49_b_ (1.11)	1.38_b_ (1.01)	1.63_b_ (1.21)	−5.39**	0.22
Depressive	1.83_c_ (1.30)	1.67_c_ (1.22)	2.02_d_ (1.38)	−6.33**	0.26
Self-perceived mate value	4.55 (1.34)	4.66 (1.32)	4.42 (1.35)	4.27**	−0.18
*Mate value indicators*					
Age	34.57 (9.37)	34.37 (8.95)	34.8 (9.84)	−1.10	0.05
Age-squared	1282.51 (648.39)	1261.28 (621.03)	1307.83 (679.02)	−1.71	0.07
Level of education	3.09 (1.25)	3.01 (1.28)	3.18 (1.20)	−3.32**	0.14
Personal income (CZK)	3.84 (2.27)	3.05 (1.83)	4.79 (2.38)	−19.69**	0.82
*Mating strategy indicators*					
*N* children	0.99 (1.07)	1.12 (1.08)	0.83 (1.03)	6.47**	−0.27
*N* relationships	7.54 (11.72)	7.08 (10.51)	8.08 (13.01)	−2.03*	0.08
*N* sex partners	8.99 (14.41)	8.46 (13.12)	9.63 (15.79)	−1.94	0.08
Relationship length (if paired)	108.14 (92.55)	108.04 (90.48)	108.27 (95.35)	−0.06	0.00
*Demographic details*					
Relationship status *N* (%)	1,851 (81.20)	1067 (86.00)	784 (75.40)	42.11**	.14
Household income (CZK)	4.87 (2.07)	4.55 (1.93)	5.27 (2.16)	−8.40**	0.35
*N* people in household	3.01 (1.26)	3.05 (1.22)	2.98 (1.31)	1.32	−0.06
Size of residence	3.52 (1.80)	3.32 (1.79)	3.76 (1.78)	−5.85**	0.25

*d* is Cohen’s *d* for effect size. *V* is Cramer’s *V* for effect size. Subscripts denote differences within desirable and undesirable factors (*p* < .05)

^a^ *df* = 2278, except in relationship length where *df* = 1849, and relationship status where *df* = 1

* *p* < .05, ** *p* < .01

First, we focused on sex differences in desirable characteristics. We tested a 2 × 7 model, revealing a sex × desirable characteristic interaction (*F*[6, 13668] = 27.92, *p* < 0.01, *η*_*p*_^*2*^ = 0.01), and sex (*F*[1, 2278] = 16.08, *p* < 0.01, *η*_*p*_^*2*^ = 0.01) and desirable characteristic main effects (*F*[6, 13668] = 382.12, *p* < 0.01, *η*_*p*_^*2*^ = 0.14). Post hoc *t* tests showed (Table 1) that women rated higher Warmth, Status, and Dominance in their mate preferences, whereas men rated higher Physical Attractiveness. Post hoc repeated-measures ANOVA showed that the preferred characteristic levels differed overall (*F*[6, 13674] = 368.68, *p* < 0.01, *η*_*p*_^2^ = 0.14), in women (*F*[6, 7434] = 151.37, *p* < 0.01, *η*_*p*_^2^ = 0.11), and in men (*F*[6, 6234] = 247.03, *p* < 0.01, *η*_*p*_^2^ = 0.19). Pairwise comparisons showed that both women and men’s most preferred characteristic was Warmth, while the least preferred was Status.

Second, we turned to undesirable characteristics. We tested a 2 × 8 model, revealing a sex × undesirable characteristic interaction (*F*[7, 15946] = 8.71, *p* < 0.01, *η*_*p*_^2^ < 0.01), and sex (*F*[1, 2278] = 36.65, *p* < 0.01, *η*_*p*_^2^ = 0.02) and undesirable characteristic main effects (*F*[7, 15946] = 1202.90, *p* < 0.01, *η*_*p*_^2^ = 0.35). Post hoc *t* tests showed (Table 1) that women wanted less Unambitious, Hostile, Arrogant, Clingy, Abusive, and Depressed partner than men did. Post hoc repeated-measures ANOVA showed that the avoidable characteristic levels differed overall (*F*[7, 15953] = 1204.98, *p* < 0.01, *η*_*p*_^2^ = 0.35), in women (*F*[7, 8673] = 636.14, *p* < 0.01, *η*_*p*_^2^ = 0.34), and in men (*F*[7, 7273] = 582.33, *p* < 0.01, *η*_*p*_^2^ = 0.36). Pairwise comparisons showed that both women and men’s least problematic characteristic was Clingy, while the most problematic were Hostile and Abusive.

Third, we turned to the correlations between mating strategy, mate value, and demographic factors in relation to desirable characteristics (Table 2) and undesirable characteristics (Table 3). When we looked for moderation by participant’s sex, we found limited evidence for moderation in correlations (of all correlations: 25% overall; 32% desirable characteristics; 18% undesirable characteristics). With the regression models, we found that overall, self-perceived mate value alone explained < 1 to 4%, mate value indicators explained < 1 to 5%, mating strategy indicators explained < 1 to 3%, demographic details explained < 1 to 3%, and all variables altogether explained 1–8% of preferences for desirable characteristics (i.e., Status, Intelligence, and Passion, in particular). Specific sex differences were found between the association with self-perceived mate value and the preference for Attractiveness (women = 6% vs men = 1%), Passion (women = 5% vs men = 1%), and Dominance (women = 5% vs. men = 2%). All variables altogether also explained women’s preferences better, in Attractiveness (women = 9% vs men = 5%), Intellect (women = 11% vs men = 6%), Passion (women = 10% vs men = 5%), except for Status (women = 7% vs men = 8%). Undesirable characteristics were explained, overall, by self-perceived mate value between < 1 and 5%, by mate value indicators between 1 and 3%, by mating strategy indicators between < 1% and 1%, by demographic variables between < 1% and 1%, and by all variables altogether between 1 and 4%. All variables altogether explained slightly better women’s undesirable characteristic levels in Unambitious (women = 4% vs men = 2%), Hostile (women = 5% vs. men = 3%), Unattractive (women = 5% vs. men = 2%), and Clingy (women = 5% vs. men = 3%).

**Table 2:** Correlations between desirable factors, self-perceived mate value, mate value indicators, mating strategy indicators, and demographic details overall, moderated by sex, and as blocks in multiple regression per trait (*R*^2^)

	Desirable factors						
	Warmth	Attractive	Status	Intelligent	Passionate	Stable	Dominant
Self-Perceived Mate Value *r* (*R*^2^)	.08** (.01**)	.18** (.03**)	.19** (.04**)	.12** (.01**)	.16** (.03**)	.01 (< .01)	.18** (.03**)
Women/Men	.11**/.03	.25**/.12**	.18**/.18**	.18**/.04	.23**/.08**	.03/-.02	.22**/.13**
Women/Men (*R*^2^)	.01**/< .01	.06**/.01**	.03**/.03**	.03**/< .01	.05**/.01**	< .01/< .01	.05**/.02**
Mate value indicators (*R*^2^)	< .01	.02**	.01**	.05**	.02**	< .01	.01**
Women/Men (*R*^2^)	.01/< .01	.03**/.02**	.01**/< .01	.07**/.03**	.03**/.01*	.01**/< .01	.02**/.01
Age	−.05*	−.14**	−.02	−.08**	−.12**	< .01	−.09**
Women/Men	−.06*/−.04	−.16**/−.13**	−.01/−.02	−.09**/−.08*	−.14**/−.09**	−.01/< .01	−.11**/−.07*
Age-squared	−.05*	−.14**	−.02	−.08**	−.12**	< .01	−.09**
Women/Men	−.06*/−.04	−.16**/−.13**	−.01/−.03	−.09**/−.07*	−.14**/−.09**	−.01/< .01	−.10**/−.07*
Level of education	.01	.05*	.06**	.19**	−.04	.04	.03
Women/Men	.03/< .01	.060/.03	.11**/.02	.24**/.13**	−.04/−.04	.10**/−.04	.06*/< .01
Personal income	−.04*	.01	−.04	.04*	−.03	< .01	−.04
Women/Men	−.03/-.01	−.02/−.03	.07*/.02	.11**/.01	< .01/-.05	.03/−.01	.01/.01
Mating strategy indicators^a^ (*R*^2^)	< .01	.02**	< .01	.01**	.03**	< .01	.01**
Women/Men^b^ (*R*^2^)	.01/.01	.02**/.02**	< .01/.02*	.02**/.01	.05**/.02**	< .01/< .01	.01*/.01
*N* children	.02	−.08**	.01	−.10**	−.02	−.01	−.03
Women/Men	−.02/.05	−.08**/−.06	−.02/−.02	−.14**/−.06	−.02/−.03	−.03/.01	−.06*/−.02
*N* relationships	.01	−.01	.03	−.01	.05*	−.01	−.03
Women/Men	−.02/.04	−.03/.01	.01/.06*	−.01/< .01	.04/.06	−.01/< .01	−.01/−.03
*N* sex partners	−.01	.03	.02	< .01	.07**	< .01	−.02
Women/Men	−.03/.01	−.01/.05	.04/.03	.01/−.01	.05/.10**	< .01/< .01	−.01/−.03
Relationship length (if paired)^a^	−.03	−.13**	−.03	−.08**	−.17**	−.01	−.09**
Women/Men^b^	−.07*/.01	−.14**/−.13**	−.01/−.06	−.10**/−.07	−.20**/−.13**	−.04/.03	−.11**/−.07*
Demographic details (*R*^2^)	.02**	.01**	.03**	.03**	.01**	< .01	.01**
Women/Men (*R*^2^)	.01**/.02**	.01**/.01*	.04**/.03**	.04**/.02**	.01**/.01**	.01*/< .01	.01**/.01
Relationship status	.11**	.07**	.12**	.06**	.11**	.03	.06**
Women/Men	.10**/.11**	.06*/.09**	.08**/.11**	.03/.10**	.10**/.11**	.01/.04	.04/.05
Household income	.06**	.07**	.10**	.13**	.03	.05*	.05*
Women/Men	.07*/.07*	.11**/.01	.18**/.10**	.17**/.11**	.06*/< .01	.09**/.02	.08**/.06*
*N* people in household	.06**	.03	−.01	−.02	.01	.01	−.01
Women/Men	.01/.12**	.03/.03	.01/−.03	−.04/.01	.02/−.01	−.01/.03	−.03/−.01
Size of residence	−.01	.02	−.02	.07**	< .01	.01	.03
Women/Men	< .01/< .01	< .01/.02	**.04/−.05**	.10**/.05	< .01/.01	.01/.01	.07*/.03
All variables, except relstatus^a^ (*R Type="Italic">*^2^)	.02**	.06**	.08**	.08**	.07**	.01	.06**
Women/Men^b^ (*R*^2^)	.03**/.02	.09**/.05**	.07**/.08**	.11**/.06**	.10**/.05**	.02*/.01	.06**/.04**

Bolded *r* values between men and women differed (*p* < .05) with a Fisher’s *z-*test; Relstatus = Relationship status

a *n* = 1851

b *n* = 1067 (women)/784 (men)

* p < .05, ** p < .01

**Table 3:** Correlations between undesirable factors, self-perceived mate value, mate value indicators, mating strategy indicators, and demographic details overall, moderated by sex, and as blocks in multiple regression per trait (*R*^2^)

	Undesirable factors							
	Unambitious	Hostile	Filthy	Arrogant	Unattractive	Clingy	Abusive	Depressive
Self-Perceived Mate Value *r* (*R*^2^)	−.02 (< .01)	.04 (< .01)	.02 (< .01)	.04* (< .01*)	−.04* (< .01*)	.05* (< .01*)	.05* (< .01*)	.01 (< .01)
Women/Men	−.02/ < .01	.02/.07*	.02/.03	.03/.07*	−.10**/.03	.05/.07*	.02/.10**	.01/.02
Women/Men (*R*^2^)	< .01/ < .01	< .01/.01*	< .01/ < .01	< .01/.01*	.01**/ < .01	< .01/ < .01*	< .01/.01**	< .01/ < .01
Mate value indicators (*R*^2^)	.01**	.02**	.01*	.01**	.02**	.03**	.02**	.02**
Women/Men (*R*^2^)	.03**/ < .01	.03**/.01*	.01**/ < .01	.01**/.01	.03**/.01	.04**/.02**	.03**/.02**	.02**/.02**
Age	.04	−.04	− .02	−.05*	.05*	−.07**	−.07**	−.05*
Women/Men	.07*/ < .01	−.03/−.06	−.01/−.04	−.02/−.07*	.10**/ < .01	−.06*/−.09**	−.02/−.12**	−.01/−.09**
Age-squared	.04	.04	−.02	−.04	.05*	−.06**	−.06**	−.04
Women/Men	.07*/ < .01	−.02/−.06	−.01/−.04	−.02/−.07*	.10**/ < .01	−.05/−.08**	−.01/−.12**	< .01/−.08**
Level of education	−.08**	−.12**	−.07**	−.07**	−.13**	−.15**	−.11**	−.09**
Women/Men	−.16**/−.01	−.17**/−.09**	−.11**/−.02	−.10**/−.04	−.16**/−.08**	−.18**/−.11**	−.16**/−.08**	−.13**/−.07*
Personal income	.01	< .01	−.04*	−.02	−.03	−.07**	−.02	−.03
Women/Men	−.07*/−.04	−.05/−.04	−.06*/−.04	−.04/−.06	−.02/−.05	−.11**/−.10**	−.04/−.09**	− .06*/ − .11**
Mating strategy indicators^a^ (*R*^2^)	< .01	< .01	< .01	< .01	.01*	< .01	.01*	< .01
Women/Men^b^ (*R*^2^)	.01/ < .01	.01/.01	< .01/ < .01	.01/.01	.02**/ < .01	< .01/.01	.01/.02**	< .01/.01
*N* children	.02	−.01	< .01	< .01	.06**	−.01	−.03	−.04
Women/Men	.06*/.01	.04/−.02	.01/ < .01	**.05/−.04**	.10**/.01	.05/−.06*	.04/−.07*	< .01/−.04
*N* relationships	−.01	.01	−.01	< .01	< .01	.01	.02	−.01
Women/Men	−.01/−.03	.03/−.02	.01/−.03	.03/−.03	.02/−.01	.01/−.01	.05/−.02	< .01/−.04
*N* sex partners	−.02	.01	−.02	< .01	−.01	< .01	− .01	−.04
Women/Men	< .01/−.05	.02/−.01	−.02/−.03	.02/−.03	.01/−.03	.03/−.03	.02/ − .04	−.01/−.08**
Relationship length (if paired)^a^	.03	−.03	−.03	−.03	.06*	−.03	−.06**	−.03
Women/Men^b^	.06*/−.02	.02/−.08*	−.01/−.05	.01/−.08*	.11**/−.01	< .01/−.09*	< .01/−.13**	−.01/−.06
Demographic details (*R*^2^)	.01**	.01**	.01**	.01**	.01**	< .01	.01**	.01**
Women/Men (*R*^2^)	.02**/.01*	.02**/.02**	.02**/.01	.01**/.01**	.02**/.02**	.01**/ < .01	.02**/.01*	.02**/.02**
Relationship status	−.04	−.08**	−.02	−.07**	−.07**	.03	−.07**	−.08**
Women/Men	−.01/−.03	−.04/−.09**	.04/−.07*	−.03/−.09**	−.04/−.10**	.07**/ < .01	−.03/−.08*	−.05/−.08*
Household income	−.08**	−.07**	−.07**	−.06**	−.08**	< .01	−.07**	−.08**
Women/Men	−.14**/−.08*	−.10**/−.07*	−.12**/−.04	− .07*/ − .08**	−.09**/−.08**	.02/−.04	−.11**/−.08*	−.12**/−.08**
*N* people in household	< .01	< .01	.01	< .01	.03	.04	.01	< .01
Women/Men	.04/−.02	**.04/−.04**	.01/.02	.06*/−.06	.05/ < .01	.09**/−.03	.04/ − .02	< .01/.01
Size of residence	.05*	−.02	.01	.02	< .01	−.01	< .01	.04
Women/Men	−.01/.07*	−.02/−.05	−.01/.02	−.01/.03	−.01/.01	−.03/ < .01	−.01/−.03	< .01/.04
All variables, except relstatus^a^ (*R*^2^)	.02**	.03**	.01	.02**	.03**	.03**	.04**	.03**
Women/Men^b^ (*R*^2^)	.04**/.02	.05**/.03	.02*/.01	.03**/.02	.05**/.02	.05**/.03*	.05**/.04**	.04**/.03*

Bolded *r* values between men and women differed (*p* < .05) with a Fisher’s *z-*test; Relstatus = Relationship status

a *n* = 1851

b *n* = 1067 (women)/784 (men)

* p < .05, ** p < .01

**Fig. 1:**
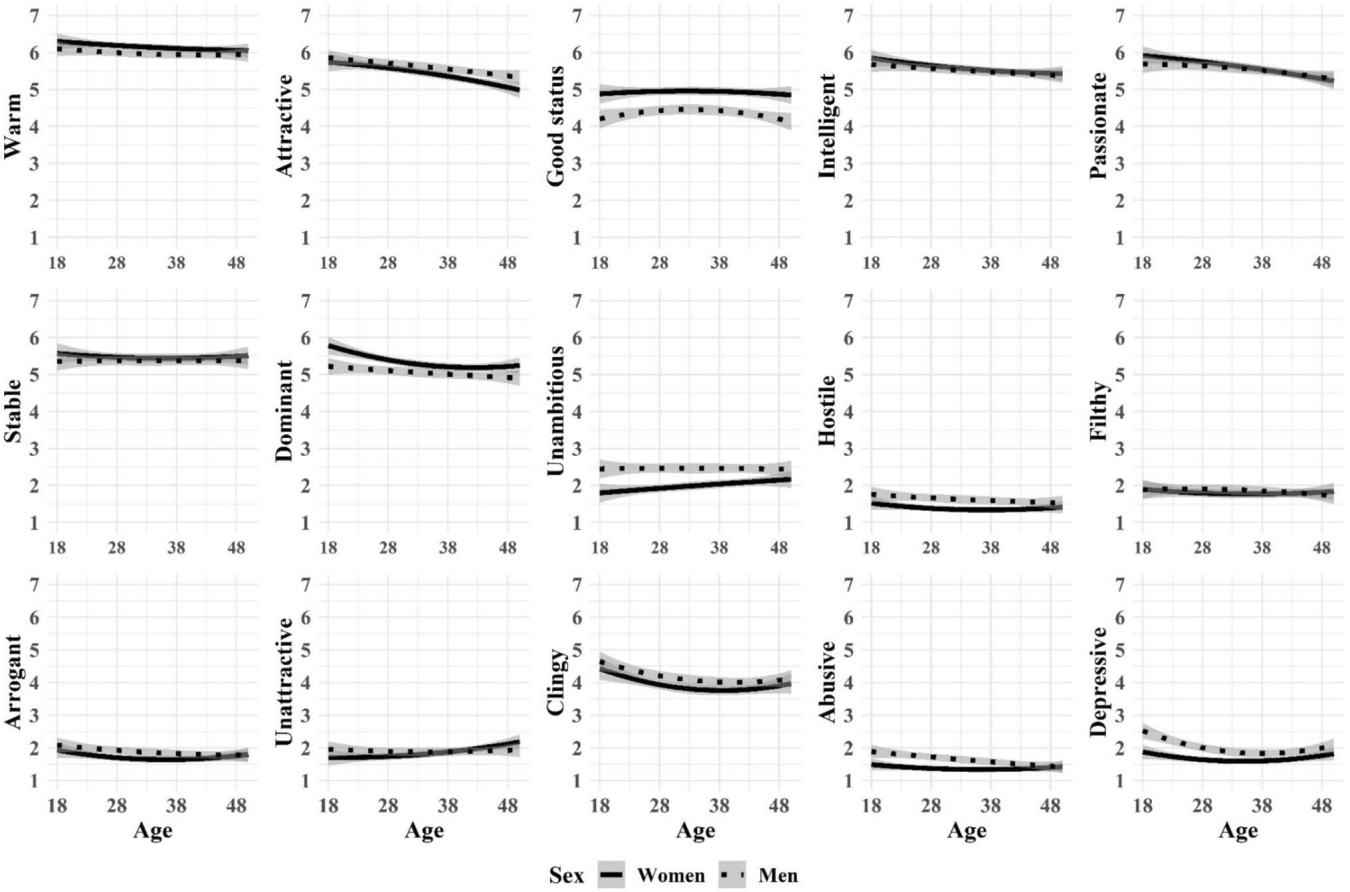
Quadratic associations (with 95% confidence intervals) between age and ideal partner preferences across men and women

**Fig. 2:**
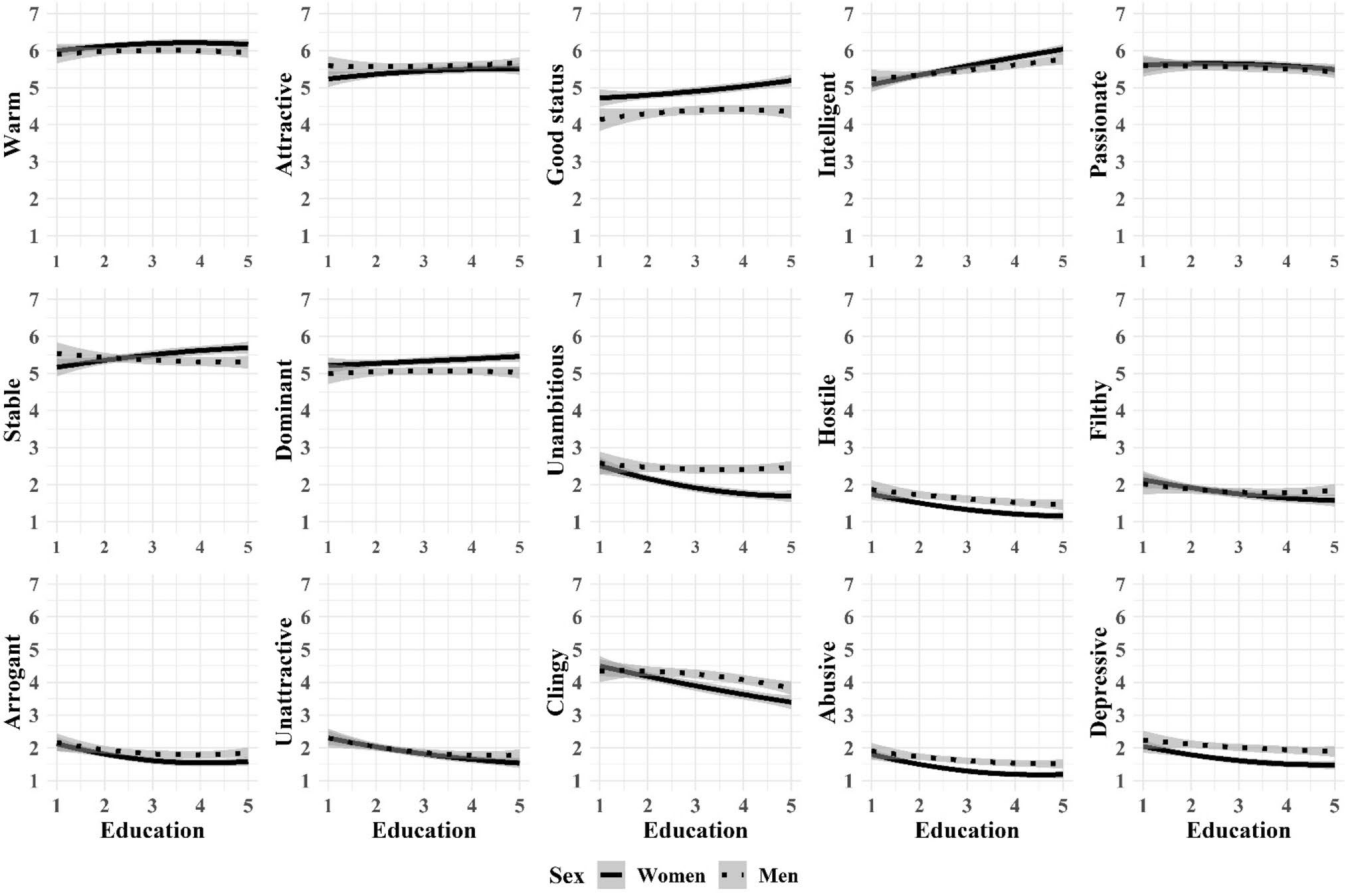
Quadratic associations (with 95% confidence intervals) between education and ideal partner preferences across men and women

**Fig. 3:**
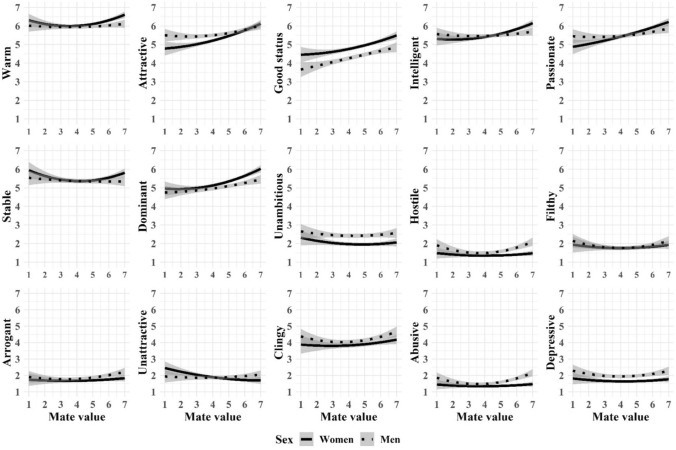
Quadratic associations (with 95% confidence intervals) between self-perceived mate value and ideal partner preferences across men and women

And last, we tried to better understand the nonlinear nature of age-effects (Fig. 1), education-effects (Fig. 2), and self-perceived mate value effects (Fig. 3) on mate preferences and aversions with curved regression analyses moderated by sex. That is, we predicted each desirable and undesirable characteristic with age (or education, or mate value), age^2^, sex, sex × age, and sex × age^2^. We found no sex × age^2^ interactions in the association between age and preferences or aversions (Fig. 1). We found curved associations without sex interaction in Dominant (*F*[5, 2274] = 10.13, *p* < 0.01, *R*^2^ = 0.02), Clingy (*F*[5, 2274] = 6.61, *p* < 0.01, *R*^2^ = 0.01), and Depressive (*F*[5, 2274] = 13.65, *p* < 0.01, *R*^2^ = 0.03). We found no sex × age^2^ interactions in the association between education and preferences or aversions (Fig. 2). We found curved associations without sex interaction in Arrogant (*F*[5, 2274] = 6.44, *p* < 0.01, *R*^2^ = 0.01) and Abusive (*F*[5, 2274] = 14.54, *p* < 0.01, *R*^2^ = 0.03). We found sex × age^2^ interactions in the association between self-perceived mate value and Stability (*F*[5, 2274] = 3.16, *p* < 0.01, *R*^2^ = 0.01), Hostility (*F*[5, 2274] = 10.58, *p* < 0.01, *R*^2^ = 0.02), and Abuse (*F*[5, 2274] = 12.69, *p* < 0.01, *R*^2^ = 0.03; Fig. 3). Further, we found curved associations without sex interaction in Warmth (*F*[5, 2274] = 8.45, *p* < 0.01, *R*^2^ = 0.02), Intellect (*F*[5, 2274] = 10.79, *p* < 0.01, *R*^2^ = 0.02), and Dominance (*F*[5, 2274] = 22.61, *p* < 0.01, *R*^2^ = 0.05).

## Discussion

In a nationally representative sample of over two thousand participants, we found that women were more demanding in their mate preferences than men in Warmth, Status, and Dominance, while men were more demanding in Physical Attractiveness than women. Women also tolerated Unambitious, Hostile, Arrogant, Clingy, Abusive, and Depressed partners less than men did. On the other hand, both sexes wanted their partners to be the highest in Warmth and the lowest in Status among the desirable characteristics, and the lowest in Hostile, and Abusive, and the highest in Clingy among the undesirable characteristics. These findings are consistent with evolutionary predictions and previous findings, even though in this study, we did not differentiate short- and long-term partners (Csajbók & Berkics, 2017, 2022; Jonason et al., 2015; Li, 2007). These results demonstrate the robustness of sex differences in desirable and undesirable characteristics across various age groups (Apostolou & Eleftheriou, 2022; Buunk et al., 2002; Greenlees & McGrew, 1994; Schwarz & Hassenbrauck, 2012).

We also found linear associations between mate preferences and aversions and self-perceived mate value, mate value indicators, mating strategy indicators, and demographic variables. Sex moderated these associations in a quarter of the correlations overall, but more likely in desirable than undesirable characteristics (32% and 18%, respectively). Self-perceived mate value alone explained desirable characteristics better than mating strategy indicators or demographic details. Self-perceived mate value also explained undesirable characteristics better than mate value indicators, mating strategy indicators, demographic variables, and all variables altogether. Overall, desirable characteristics were better explained (3% on average) by all subjective and objective mate value indicators than undesirable characteristics were (1% on average). The prediction of mate preferences and aversions by mate value and its indicators was better in women in virtually all factors except for Status.

All in all, these findings were in accordance with previous research where the dominance of self-perceived mate value over objective mate value indicators was demonstrated (Csajbók et al., 2019, 2023). Interestingly, sex differences in these associations were consistent with the predictions. That is, women with more mate value were more demanding in their mate preferences, while the association between mate value indicators and preferences was not pronounced in men (Regan, 1998).

We studied nonlinear relationships between age, education, self-perceived mate value, and mate preferences and aversions. While age is a frequently studied and assumed predictor of mate preference changes over the lifespan (Brumbaugh & Wood, 2013; Fales et al., 2016; Sprecher et al., 2019), we found trivial associations with age (explained variances ranged between < 1% and 3%) even when considering curved and sex-moderated associations too. The tiny change in preferences between 18 and 50 years is rather surprising. Younger (fertile) people have long been believed to be pickier than older individuals. From a reproductive value perspective (Buss et al., 2000), fertility, especially in women, tends to decline. Consequently, the urgency to find a highly fertile and high-quality partner is reduced.

One might also expect that previous relationships and life experiences could somewhat shape mate preferences (Brumbaugh & Wood, 2013). Furthermore, older individuals may have achieved greater socioeconomic stability over time, reducing the need to seek a partner for financial security (Stanik & Ellsworth, 2010). This can lead to the focus on other qualities in mate selection. However, our results demonstrate that age plays a negligible role not only in partner preferences (e.g., Schwarz & Hassebrauck, 2012) but also in aversions (Apostolou & Eleftheriou, 2022). Meanwhile, education showed a stronger association with desirable and undesirable characteristics than age (explained variances ranged between < 1% and 4%), but these associations were more likely linear. The most pronounced nonlinear relationships were found with self-perceived mate value (explained variances ranged between < 1% and 5%).

Concerning objective mate value indicators more specifically, mate value was negatively correlated with age—albeit weakly—and positively correlated with mating standards in previous research (Csajbók et al., 2019), just like here, although the association of mate value with undesirable characteristics was more mixed than with desirable characteristics (Csajbók et al., 2019; Csajbók & Berkics, 2022). Being in a relationship and having more sex partners (thus having a short-term mating orientation) were positive predictors of higher mating standards in Passion and lower in Depression. Understandably, those who are valued by someone else and have more mating success (i.e., sex partners) will increase their self-perceived mating potential and, thus, their mating expectations as well (Brase & Guy, 2004; Csajbók et al., 2019). On the other hand, the longer their relationships were, the more our participants relaxed their standards in Attractiveness, Passion, Dominance, and women in Warmth and Intellect too. People probably adjusted their expectations as they gained more relationship experience, or after they entered a relationship, they adjusted their ideals to the realities of being in a relationship (Gerlach et al., 2019; Kučerová et al., 2018).

Also, the more children they had, the more women relaxed their standards in Attractiveness, Intellect, Dominance, Unambitious, and Unattractive. This is an interesting finding considering the association with the length of the relationships, as both mating strategy indicators can be associated with long-term mating orientation. Further, the number of sex partners, a short-term mating strategy indicator, was also associated with more instead of less mating expectations. However, people are often more demanding in their long-term partner preferences than in their short-term ideals, except for Physical Attractiveness (e.g., Csajbók & Berkics, 2017; Li, 2007). One possible reason for our contradictory result was that we did not specify for the participants what kind of relationship they should indicate their ideal partner for. We did so to avoid suggesting artificial relationship types (such as short-term or long-term relationships) which might have low external validity in the participants’ eyes (Marzoli et al., 2018). We assume the participants indicated their ideal partners for their ideal relationships—may they be casual or committed.

Interestingly, we also found sex differences in the associations with education and household income. For example, both men and women preferred a more Intelligent partner if they themselves were also more educated (Eika et al., 2019; Mare, 1991), but especially educated women wanted an intelligent partner with good earning capacity (Status) and emotional Stability. Women also accepted less Unambitious, Hostile, Filthy, Unattractive, Abusive, Hostile, and Depressed partners if they had more education, while men’s preferences were less pronounced for these characteristics.

Similarly, more household income was correlated with higher expectations for desirable and lower for undesirable characteristics, especially in women, which effects are like those with education and intelligence (Jonason & Antoon, 2019; Jonason et al., 2019). This is especially interesting from the perspective that personal income did not correlate with expectations in as many factors or as strongly as household income did. This finding could indicate that members of wealthy households experience an amplified mate value increase as they jointly make a good income. This advantage might lead to the romantic equivalent of “power couples” who experience additional benefits from both having good education and high earning capacity (Florida et al., 2022). Thus, couples in a high-earning household have not only better living and working conditions but also an increased sense of mate choice power.

Overall, unexpectedly, men’s preferences did not seem to correlate as strongly with their sociodemographic potential as women’s preferences did, although women’s higher preferences for status and resources in men are widely reported (e.g., Buss, 1989; Kenrick et al., 1993; Li, 2007). In other words, while women value men’s status more than men value women’s status in the mating context (Jonason et al., 2012), men with lower status indicators did not decrease their preferences (because of lower mate choice power) as much as women did. This is surprising because women are more dependent than men on their environment’s help to acquire resources and shelter while taking care of a baby (Trivers, 1972). Our results show overall that women’s mate value and thus mate choice power were better predicted by these indicators than men’s were.

The takeaway is that demographic characteristics do not predict mate preferences, and relatedly, do not approximate mate value well. While we often assume that people with more money, higher education, and fertile age would have higher mate value and thus higher preferences, we can see that with sufficient power and diversity in the sample, these associations are weak. They are in the expected direction, however. We suggest that the likely true magnitude of these associations should be considered when researchers hypothesize and explain certain assumptions about demographics influencing mate choice. It is often assumed that we observe certain evolutionary anomalies, for example, status being the least important factor among many (as it was found in the current study, and also in e.g., Fletcher et al., 1999; Katsena & Dimdins, 2015), because the sample is not “poor” enough (e.g., Li et al., 2013). If this was the case, we could argue that we should have seen at least slightly stronger results here where the sample was more balanced in this regard.

Alternatively, these findings can be attributed to the measurement choice. Here, we used the simple rating method, but there is research that indicates that simple rating measures may not have good predictive validity (e.g., Eastwick et al., 2019), or in every situation (e.g., Li et al., 2013). An alternative measurement method would be to rely on trade-off models, for example with the use of budget allocation methods (e.g., Li, 2007). The advantage of trade-off methods, in comparison to the simple rating methods, is that they constrain the participants to differentiate necessities from luxuries. However, recent findings on a large sample of Hungarian participants found weaker individual differences effects (e.g., age, education, number of short- and long-term romantic relationships, mate value measured by the Mate Value Scale, Edlund & Sagarin, 2014) on budget allocation data than on simple rating data (Shweiki, 2024). Mate preference integration is also an aspect that is not independent of how we regard the individual role of each mate preference and aversion factor (e.g., Conroy-Beam, 2018). That is, the combination of these preference factors and the level to which people fair on them influences the overall Gestalt of mate choice in ways which were not considered in this study.

### Limitations and Conclusions

Although our sample was collected following nationally representative quotas, the final sample, after cleaning, differed from the set quotas in sex, age, size of residential area, and education. Still, the age difference from the quotas was beneficial for us. The current study could operate with more older people (45–50 years of age) than the general population, who are often hard to reach and are underrepresented in psychological research. On the other hand, even though the sample was diverse within the studied population, our sample can be still considered WEIRD (Henrich et al., 2010).

Also, our study tested associations between mate preferences and age cross-sectionally; thus, our interpretation in attributing age differences to changes over the lifespan is tentative and limited. Some of the variance observed in connection to age may be cohort effects and the result of unaccounted for historical, social, and cultural factors. Future research should focus on longitudinal changes in a within-subject design so that we can better interpret and attribute the source of longitudinal changes in mate preferences (e.g., Driebe et al., 2023).

Also, while a simple, face-valid form of mate value measure that we used here is assumed to have better predictive validity by some (e.g., Edlund & Sagarin, 2014; Sela et al., 2017) than more complex psychometric approaches (e.g., Fisher et al., 2008), our measure was arguably limited. In other words, we cannot be certain participants understood our single-item, self-perceived mate value measure as intended. Still, it is more and more common that researchers rely on parsimonious, single-item measures for their benefits, such as brevity and face validity (e.g., Allen et al., 2022). Also, the magnitude of the correlations between mate value and mate preferences varied a lot in previous research, depending on measures used and sample size (e.g., Arnocky, 2018), and our effect sizes are comparable with other large sample size studies using multiple-item measures (Csajbók et al., 2019; Csajbók & Berkics, 2022). Lastly, as we noted above, we did not differentiate between long- and short-term partner ideals because our strategy was to avoid creating an artificial dichotomy and because we aimed to keep the survey succinct. There is ample evidence of differences in partner preferences when such distinctions are made, but still, several sex differences were also demonstrated here consistent with previous research without such contextual differentiations.

In this study, we tested the hypothesis that men and women increase their mate preferences while having more subjective and objective mate value indicators. We also studied how preferences correlated with mating strategy orientation. Our participants were expected to have higher expectations for desirable and lower for undesirable characteristics as they thought they were more desirable, and as they had more objective mate value, long-term mating strategy indicators, and better demographic features. The underlying rationale was that men attract a mate by offering resources, while women can afford to be pickier as they possess more desirable characteristics signaling youth and fertility. In a nationally representative Czech sample with a diverse population from all residential areas, levels of education, sex, and age categories, we found that all demographic, mate value, and mating strategy characteristics explained, on average, 3% of men’s and 5% of women’s preferences. Meanwhile, self-perceived mate value alone explained on average 1% of men’s and 2% of women’s preferences across 15 desirable and undesirable factors.

The magnitude of these associations was dependent on the individual characteristics measured. Desirable factors were better explained (3% on average) by the participants’ characteristics than undesirable factors were (1% on average). We also found some evidence for nonlinear associations between preferences and aversions and age, education, and self-perceived mate value. Overall, we conclude that objective mate value indicators such as age, education, income, number and length of relationships, and other demographic variables predict mate preferences and aversions; however, other factors, such as self-perceived mate value, may be more strongly associated with mate preferences. Nevertheless, the factors accounting for over 90% of the remaining variability in mate preferences and aversions remain unclear.

## Supplementary Information

Below is the link to the electronic supplementary material.

Supplementary file1 (DOCX 57 KB)

## Data Availability

This study was not preregistered. The data that support the findings of this study and R-codes are available on the Open Science Framework https://osf.io/2unwy/?view_only=3a7e20ef406f4dd084de0675dcc437e6.

## Footnotes

1 See the complete list of literature reviewed on age associated with mate preferences in Supplementary Table S1.

2 https://nationalpanel.eu/?lang=en

3 We did so to ensure that the sex of the ideal partner aligns within all men and all women.

4 The final sample differed from the general population in age, size of the residential area, and the highest level of education (Supplementary Table S2), which is a natural consequence of the reliance on the online data collection method (see Supplementary Methods).

5 Our intention was to measure desirability, but the Czech equivalent of this expression (přitažlivý) has a meaning somewhere between attractive and desirable.

6 Participants also rated themselves and their partners on the same 15 factors in random order, but these ratings were not used here.

## References

[CR1] Allen, M. S., Iliescu, D., & Greiff, S. (2022). Single item measures in psychological science. *European Journal of Psychological Assessment,**38*, 1–5.

[CR2] Al-Shawaf, L., Lewis, D. M., Ghossainy, M. E., & Buss, D. M. (2019). Experimentally inducing disgust reduces desire for short-term mating. *Evolutionary Psychological Science,**5*, 267–275.

[CR3] Apostolou, M., & Eleftheriou, C. (2022). What constitutes bad flirting: An explorative study of dealbreakers. *Personality and Individual Differences,**194*, 111665.

[CR4] Arnocky, S. (2018). Self-perceived mate value, facial attractiveness, and mate preferences: Do desirable men want it all? *Evolutionary Psychology,**16*, 1474704918763271. 29534596 10.1177/1474704918763271 PMC10480998

[CR5] Brase, G. L., & Guy, E. C. (2004). The demographics of mate value and self-esteem. *Personality and Individual Differences,**36*, 471–484.

[CR6] Brumbaugh, C. C., & Wood, D. (2013). Mate preferences across life and across the world. *Social Psychological and Personality Science,**4*, 100–107.

[CR7] Buss, D. M. (1989). Sex differences in human mate preferences: Evolutionary hypotheses tested in 37 cultures. *Behavioral and Brain Sciences,**12*, 1–14.

[CR8] Buss, D. M., & Schmitt, D. P. (1993). Sexual strategies theory: An evolutionary perspective on human mating. *Psychological Review,**100*, 204–232. 8483982 10.1037/0033-295x.100.2.204

[CR9] Buss, D. M., & Schmitt, D. P. (2019). Mate preferences and their behavioral manifestations. *Annual Review of Psychology,**70*, 77–110. 30230999 10.1146/annurev-psych-010418-103408

[CR10] Buss, D. M., Shackelford, T. K., & LeBlanc, G. J. (2000). Number of children desired and preferred spousal age difference: Context-specific mate preference patterns across 37 cultures. *Evolution and Human Behavior,**21*, 323–331. 10.1016/s1090-5138(00)00048-9 11053693

[CR11] Buunk, B. P., Dijkstra, P., Fetchenhauer, D., & Kenrick, D. T. (2002). Age and gender differences in mate selection criteria for various involvement levels. *Personal Relationships,**9*, 271–278.

[CR12] Carton, H., & Egan, V. (2017). The dark triad and intimate partner violence. *Personality and Individual Differences,**105*, 84–88.

[CR13] Conroy-Beam, D. (2018). Euclidean mate value and power of choice on the mating market. *Personality and Social Psychology Bulletin,**44*, 252–264. 29082804 10.1177/0146167217739262

[CR14] Csajbók, Z., & Berkics, M. (2017). Factor, factor, on the whole, who’s the best fitting of all?: Factors of mate preferences in a large sample. *Personality and Individual Differences,**114*, 92–102.

[CR15] Csajbók, Z., & Berkics, M. (2022). Seven deadly sins of potential romantic partners: The dealbreakers of mate choice. *Personality and Individual Differences,**186*, 111334.

[CR16] Csajbok, Z., Havlíček, J., Demetrovics, Z., & Berkics, M. (2019). Self-perceived mate value is poorly predicted by demographic variables. *Evolutionary Psychology,**17*, 1474704919829037. 30816069 10.1177/1474704919829037 PMC10481051

[CR17] Csajbók, Z., Štěrbová, Z., Brewer, G., Cândea, C. A., De Backer, C. J. S., Fernández, A. M., Fisher, M. L., Garcia, J. R., Kruger, D. J., Massar, K., Oberzaucher, E., Quintelier, K. J. P., van Geffen, R. E., Varella Valentova, J., Varella, M. A. C., & Jonason, P. K. (2023). Individual differences in how desirable people think they are as a mate. *Archives of Sexual Behavior,**52*, 2475–2490. 37154879 10.1007/s10508-023-02601-x PMC10501943

[CR18] Csajbók, Z., Štěrbová, Z., Jonason, P. K., Cermakova, P., Dóka, Á., & Havlíček, J. (2022). Variation in depressive symptom trajectories in a large-scale sample of couples. *Translational Psychiatry**12*, 206–211. 35581177 10.1038/s41398-022-01950-w PMC9113986

[CR19] De Sousa Campos, L., Otta, E., & De Oliveira Siqueira, J. (2002). Sex differences in mate selection strategies: Content analyses and responses to personal advertisements in Brazil. *Evolution and Human Behavior,**23*, 395–406.

[CR20] Driebe, J. C., Stern, J., Penke, L., & Gerlach, T. M. (2023). Stability and change of individual differences in ideal partner preferences over 13 years. *Personality and Social Psychology Bulletin,**50*(8), 1263–1279. 37029599 10.1177/01461672231164757 PMC11193321

[CR21] Eastwick, P. W., Finkel, E. J., & Simpson, J. A. (2019). Best practices for testing the predictive validity of ideal partner preference-matching. *Personality and Social Psychology Bulletin,**45*, 167–181. 29947571 10.1177/0146167218780689

[CR22] Edlund, J. E., & Sagarin, B. J. (2014). The Mate Value Scale. *Personality and Individual Differences,**64*, 72–77.

[CR23] Eika, L., Mogstad, M., & Zafar, B. (2019). Educational assortative mating and household income inequality. *Journal of Political Economy,**127*, 2795–2835.

[CR24] Evans, K., & Brase, G. L. (2007). Assessing sex differences and similarities in mate preferences: Above and beyond demand characteristics. *Journal of Social and Personal Relationships,**24*, 781–791.

[CR25] Fales, M. R., Frederick, D. A., Garcia, J. R., Gildersleeve, K. A., Haselton, M. G., & Fisher, H. E. (2016). Mating markets and bargaining hands: Mate preferences for attractiveness and resources in two national U.S. studies. *Personality and Individual Differences,**88*, 78–87.

[CR26] Fisher, M., Cox, A., Bennett, S., & Gavric, D. (2008). Components of self-perceived mate value. *Journal of Social, Evolutionary, and Cultural Psychology,**2*, 156–168.

[CR27] Fletcher, G. J., Simpson, J. A., Campbell, L., & Overall, N. C. (2015). Pair-bonding, romantic love, and evolution: The curious case of *Homo sapiens*. *Perspectives on Psychological Science,**10*, 20–36. 25910380 10.1177/1745691614561683

[CR28] Fletcher, G. J., Simpson, J. A., Thomas, G., & Giles, L. (1999). Ideals in intimate relationships. *Journal of Personality and Social Psychology,**76*, 72–89. 9972554 10.1037//0022-3514.76.1.72

[CR29] Florida, R., Mellander, C., & King, K. (2022). Power couples, cities, and wages. *Environment and Planning A: Economy and Space,**54*, 1236–1255.

[CR30] Furnham, A. (2009). Sex differences in mate selection preferences. *Personality and Individual Differences,**47*, 262–267.

[CR31] Gerlach, T. M., Arslan, R. C., Schultze, T., Reinhard, S. K., & Penke, L. (2019). Predictive validity and adjustment of ideal partner preferences across the transition into romantic relationships. *Journal of Personality and Social Psychology,**116*, 313–330. 28921999 10.1037/pspp0000170

[CR32] Greenlees, I. A., & McGrew, W. C. (1994). Sex and age differences in preferences and tactics of mate attraction: Analysis of published advertisements. *Ethology and Sociobiology,**15*, 59–72.

[CR33] Hammen, C. L., & Peters, S. D. (1977). Differential responses to male and female depressive reactions. *Journal of Consulting and Clinical Psychology,**45*, 994–1001. 925245 10.1037//0022-006x.45.6.994

[CR34] Hammen, C. L., & Peters, S. D. (1978). Interpersonal consequences of depression: Responses to men and women enacting a depressed role. *Journal of Abnormal Psychology,**87*, 322–332. 681603

[CR35] Henrich, J., Heine, S. J., & Norenzayan, A. (2010). The weirdest people in the world? *Behavioral and Brain Sciences,**33*, 61–83. 20550733 10.1017/S0140525X0999152X

[CR36] Jonason, P. K., & Antoon, C. N. (2019). Mate preferences for educated partners: Similarities and differences in the sexes depend on mating context. *Personality and Individual Differences,**148*, 57–61.

[CR37] Jonason, P. K., Garcia, J., Webster, G. D., Li, N. P., & Fisher, H. (2015). Relationship dealbreakers: What individuals do not want in a mate. *Personality and Social Psychological Bulletin,**41*, 1697–1711. 10.1177/0146167215609064 26445853

[CR38] Jonason, P. K., Li, N. P., & Madson, L. (2012). It’s not all about the *Benjamins*: Understanding preferences for mates with resources. *Personality and Individual Differences,**52*, 306–310.

[CR39] Jonason, P. K., Li, N. P., Webster, G. W., & Schmitt, D. P. (2009). The Dark Triad: Facilitating short-term mating in men. *European Journal of Personality,**23*, 5–18.

[CR40] Jonason, P. K., Marsh, K., Dib, O., Plush, D., Doszpot, M., Fung, E., Crimmins, K., Drapski, M., & Di Pietro, K. (2019). Is smart sexy?: Examining the role of relative intelligence in mate preferences. *Personality and Individual Differences,**139*, 53–59.

[CR41] Jonason, P. K., & Thomas, A. G. (2022). Being more educated and earning more increases romantic interest: Data from 1.8M online daters from 24 nations. *Human Nature,**33*, 115–131. 35380344 10.1007/s12110-022-09422-2 PMC9250459

[CR42] Katsena, L., & Dimdins, G. (2015). An improved method for evaluating ideal standards in self-perception and mate preferences. *Scandinavian Journal of Psychology,**56*, 228–235. 25580983 10.1111/sjop.12186

[CR43] Kenrick, D. T., Gabrielidis, C., Keefe, R. C., & Cornelius, J. S. (1996). Adolescents’ age preferences for dating partners: Support for an evolutionary model of life-history strategies. *Child Development,**67*, 1499–1511. 8890497

[CR44] Kenrick, D. T., Groth, G. E., Trost, M. R., & Sadalla, E. K. (1993). Integrating evolutionary and social exchange perspectives on relationships: Effects of gender, self-appraisal, and involvement level on mate selection criteria. *Journal of Personality and Social Psychology,**64*, 951–969.

[CR45] Kenrick, D. T., & Keefe, R. C. (1992). Age preferences in mates reflect sex differences in human reproductive strategies. *Behavioral and Brain Sciences,**15*, 75–91.

[CR46] Kirsner, B. R., Figueredo, A. J., & Jacobs, W. J. (2003). Self, friends, and lovers: Structural relations among Beck Depression Inventory scores and perceived mate values. *Journal of Affective Disorders,**75*, 131–148. 12798253 10.1016/s0165-0327(02)00048-4

[CR47] Kučerová, R., Csajbók, Z., & Havlíček, J. (2018). Coupled individuals adjust their ideal mate preferences according to their actual partner. *Personality and Individual Differences,**135*, 248–257.

[CR48] Li, N. P. (2007). Mate preference necessities in long-and short-term mating: People prioritize in themselves what their mates prioritize in them. *Acta Psychologica Sinica,**39*, 528–535.

[CR49] Li, N. P., Yong, J. C., Tov, W., Sng, O., Fletcher, G. J., Valentine, K. A., & Balliet, D. (2013). Mate preferences do predict attraction and choices in the early stages of mate selection. *Journal of Personality and Social Psychology,**105*, 757–776. 23915041 10.1037/a0033777

[CR50] Li, P. F., & Johnson, L. N. (2018). Couples’ depression and relationship satisfaction: Examining the moderating effects of demand/withdraw communication patterns. *Journal of Family Therapy,**40*, S63–S85.

[CR51] Lim, G. Y., Tam, W. W., Lu, Y., Ho, C. S., Zhang, M. W., & Ho, R. C. (2018). Prevalence of depression in the community from 30 countries between 1994 and 2014. *Scientific Reports,**8*, 2861–2871. 29434331 10.1038/s41598-018-21243-x PMC5809481

[CR52] Little, A. C., DeBruine, L. M., & Jones, B. C. (2011). Exposure to visual cues of pathogen contagion changes preferences for masculinity and symmetry in opposite-sex faces. *Proceedings of the Royal Society B: Biological Sciences,**278*, 2032–2039. 10.1098/rspb.2010.1925 PMC3107643 21123269

[CR53] Luo, S. (2017). Assortative mating and couple similarity: Patterns, mechanisms, and consequences. *Social and Personality Psychology Compass,**11*, e12337.

[CR54] Mare, R. D. (1991). Five decades of educational assortative mating. *American Sociological Review,**56*, 15–32.

[CR55] Marzoli, D., Havlíček, J., & Roberts, S. C. (2018). Human mating strategies: From past causes to present consequences. *Wiley Interdisciplinary Reviews: Cognitive Science*, *9*, e1456. 10.1002/wcs.1456 28906068

[CR56] Moore, F., Cassidy, C., & Perrett, D. I. (2010). The effects of control of resources on magnitudes of sex differences in human mate preferences. *Evolutionary Psychology,**8*, 720–735. 22947829 10.1177/147470491000800412 PMC10480865

[CR57] Pflüger, L. S., Oberzaucher, E., Katina, S., Holzleitner, I. J., & Grammer, K. (2012). Cues to fertility: Perceived attractiveness and facial shape predict reproductive success. *Evolution and Human Behavior,**33*, 708–714.

[CR58] Regan, P. C. (1998). What if you can’t get what you want?: Willingness to compromise ideal mate selection standards as a function of sex, mate value, and relationship context. *Personality and Social Psychology Bulletin,**24*, 1294–1303.

[CR59] Schmitt, D. P. (2005). Sociosexuality from Argentina to Zimbabwe: A 48-nation study of sex, culture, and strategies of human mating. *Behavioral and Brain Sciences,**28*, 247–275. 16201459 10.1017/s0140525x05000051

[CR60] Schmitt, D. P. (2007). Sexual strategies across sexual orientations: How personality traits and culture relate to sociosexuality among gays, lesbians, bisexuals, and heterosexuals. *Journal of Psychology & Human Sexuality,**18*, 183–214.

[CR61] Schwarz, S., & Hassebrauck, M. (2012). Sex and age differences in mate-selection preferences. *Human Nature,**23*, 447–466. 22941269 10.1007/s12110-012-9152-x

[CR62] Sela, Y., Mogilski, J. K., Shackelford, T. K., Zeigler-Hill, V., & Fink, B. (2017). Mate value discrepancy and mate retention behaviors of self and partner. *Journal of Personality,**85*, 730–740. 27542990 10.1111/jopy.12281

[CR63] Shweiki, O. (2024). *Individual differences in necessities and luxuries in mate choice.* [Bachelor’s thesis, Charles University, Prague]. Charles University Digital Repository. https://dspace.cuni.cz/handle/20.500.11956/190938

[CR64] Singh, D. (2002). Female mate value at a glance: Relationship of waist-to-hip ratio to health, fecundity and attractiveness. *Neuroendocrinology Letters,**23*, 81–91. 12496738

[CR65] Sprecher, S., Econie, A., & Treger, S. (2019). Mate preferences in emerging adulthood and beyond: Age variations in mate preferences and beliefs about change in mate preferences. *Journal of Social and Personal Relationships,**36*, 3139–3158.

[CR66] Stanik, C. E., & Ellsworth, P. C. (2010). Who cares about marrying a rich man?: Intelligence and variation in women’s mate preferences. *Human Nature,**21*, 203–217.

[CR67] Thomas, A. G., Jonason, P. K., Blackburn, J. D., Kennair, L. E. O., Lowe, R., Malouff, J., & Li, N. P. (2020). Mate preference priorities in the East and West: A cross-cultural test of the mate preference priority model. *Journal of Personality,**88*, 606–620. 31494937 10.1111/jopy.12514

[CR68] Trivers, R. (1972). Parental investment and sexual selection. In B. Campbell (Ed.), *Sexual selection and the descent of Man* (pp. 136–179). Berlin: Aldine de Gruyter.

[CR69] Walter, K. V., Conroy-Beam, D., Buss, D. M., Asao, K., Sorokowska, A., Sorokowski, P., Aavik, T., Akello, G., Alhabahba, M. M., Alm, C., Amjad, N., Anjum, A., Atama, C. S., Atamtürk Duyar, D., Ayebare, R., Batres, C., Bendixen, M., Bensafia, A., Bizumic, B., Boussena, M., … Zupančič, M. (2020). Sex differences in mate preferences across 45 countries: A large-scale replication. *Psychological Science,**31*, 408–423. 10.1177/0956797620904154 32196435

[CR70] Watson, D., Beer, A., & McDade-Montez, E. (2014). The role of active assortment in spousal similarity. *Journal of Personality,**82*, 116–129. 23551151 10.1111/jopy.12039

