# Supplementary material for: Observed Aspects of Mate Value and Sociosexuality Account for Mate Preferences: Data from a Large, Representative Study from Czechia
Source: Arch Sex Behav. 2024 Oct 21;54(1):309–22. doi: 10.1007/s10508-024-03010-4 (PMC11782345; doi:10.1007/s10508-024-03010-4)
Supplement: Supplementary file 1 — Supplementary file1 (DOCX 57 KB) [file 10508_2024_3010_MOESM1_ESM.docx]

# Observed aspects of mate value and sociosexuality account for mate preferences:

**Data from a large, representative study from Czechia**

# SUPPLEMENTARY MATERIALS

| Supplementary Table 1. Main characteristics of studies testing age-related effect on partner preferences in chronological order | | | | | | | |
| --- | --- | --- | --- | --- | --- | --- | --- |
|  | Study | N (Female/Male); age range (mean, SD) | Age variable coding | Type of used analysis | Context | Characteristics | Effect size (Female/Male) |
| 1 | South, 1991+ | 920/1091; 18-35 years (M=24.3, SD=4.8) | Continuous | Linear regression (*β*) | Importance | Older by 5+ years | .002/.017 |
|  |  |  |  |  |  | Younger by 5+ years | .096**/.076** |
|  |  |  |  |  |  | Married before | .070**/.111** |
|  |  |  |  |  |  | With children | .073**/.097** |
|  |  |  |  |  |  | Unlikely to hold a job | .008/.038** |
|  |  |  |  |  |  | Different region | -.002/.028 |
|  |  |  |  |  |  | Different race | -.001/.042** |
|  |  |  |  |  |  | Not good-looking | -.017/.054** |
|  |  |  |  |  |  | Earing much more | -.007/-.003 |
|  |  |  |  |  |  | Earing much less | -.022/.026 |
|  |  |  |  |  |  | More education | -.022/.015 |
|  |  |  |  |  |  | Less education | -.017/.008 |
| 2 | Buunk et al., 2002 | 67/70; 20-60 years; (?, ?) | 20-29; 30-39; 40-49; 50-59 | ANOVA | Preferences | income, education, physical attractiveness, self-confidence, intelligence, social position, and dominance | ? |
| 3 | Tadinac & Hromatko, 2007 | 885/639; 16-58 years (M=26.9, ?) | <24; 25-39; 40+ |  | Importance | Good cook and housekeeper | ? |
|  |  |  |  |  |  | Pleasing disposition | ? |
|  |  |  |  |  |  | Similar education | ? |
|  |  |  |  |  |  | Refinement and neatness | ? |
|  |  |  |  |  |  | Good financial prospect | ? |
|  |  |  |  |  |  | Chastity | ? |
|  |  |  |  |  |  | Dependable character | ? |
|  |  |  |  |  |  | Emotional stability and maturity | ? |
|  |  |  |  |  |  | Desire for home and children | ? |
|  |  |  |  |  |  | Favorable social status | ? |
|  |  |  |  |  |  | Good looks | ? |
|  |  |  |  |  |  | Similar religious background | ? |
|  |  |  |  |  |  | Ambition and industrious | ? |
|  |  |  |  |  |  | Similar political background | ? |
|  |  |  |  |  |  | Mutual attraction-love | ? |
|  |  |  |  |  |  | Good health | ? |
|  |  |  |  |  |  | Education and intelligence | ? |
| 4 | Wood & Brumbaugh, 2009 | 1371 (M=26.4, SD=9.1)/2680 (M=27.8, SD=10.1); range? | Continuous | Correlation (*r*) | Preferences | Suggestive (vs modest) | -.12*/-.10* |
|  |  |  |  |  |  | Curvaceous (F)/toned (M) | .01/.00 |
|  |  |  |  |  |  | Confident | -.04/-.04 |
|  |  |  |  |  |  | Feminine (F)/masculine (M) | .03/.05 |
|  |  |  |  |  |  | Conventional (vs. countercultural) | .21*/.34* |
|  |  |  |  |  |  | Softhearted, sensitive | .16*/.16* |
|  |  |  |  |  |  | Formal, classy | .15*/.26* |
|  |  |  |  |  |  | Intelligent, smart | .09*/.22* |
|  |  |  |  |  |  | Well-groomed (vs. unkempt) | .06*/.17* |
|  |  |  |  |  |  | Trendy, stylish, urban | -.10*/-.15* |
|  |  |  |  |  |  | Smile | .25*/.15* |
|  |  |  |  |  |  | Thin | -.02/-.19* |
| 5 | Fitzpatrick et al., 2009+ | 2100/1401; 40-69 years (?, ?) | 40-49; 50-59; 60-69 | Chi-square test | Willingness to date | Different race/ethnicity | ? |
|  |  |  |  |  |  | Different religion | ? |
|  |  |  |  |  |  | Had much less money | ? |
| 6 | Alterovitz & Mendelsohn, 2009 | 300/300; 20-75+ years | Continuous | Correlation (*r*) | Preference | Age | 0.97/0.95 |
|  |  |  | 20-34 (M 27.6; SD 4.03)  40-54 (M 46.2; SD 4.16)  60-74 (M 63.6; SD 3.49)  75+ (M 7.77; SD 2.95) | ANOVA |  | Social status | ? |
|  |  |  |  |  |  | Physical attractiveness | ? |
|  |  |  |  |  |  | Selectivity (based on17 items. e.g., hair color, ethnicity, and smoking habits, as well as the questions about status and body type) | ? |
| 7 | McIntosh et al., 2011 | 46 (M=29.00, SD=3.21)/48 (M=30.08, SD=3.23); 25-35 years | Younger 25-35  Older 65+ | ANOVA (*η*^2^) | Acceptance | Youngest acceptable age | .39*** |
|  |  | 43 (M=67.77, SD=2.75)/38 (M=69.24, SD=5.20); 65+ years |  |  |  | Oldest acceptable age | .16*** |
|  |  |  |  |  |  | Shortest acceptable height | .03* |
|  |  |  |  |  |  | Lowest acceptable Income | .02* |
|  |  |  |  |  |  | Willingness to date interracially | .08*** |
|  |  |  |  |  |  | Willingness to date other religions | .05** |
|  |  |  |  |  |  | Distance | .06** |
| 8 | Schwarz & Hassebrauck, 2012 | 21245; 18-65 years (M=41.16, SD=10.54) | Continuous | Hierarchical regression analyses (*r*) | Importance | Creative and domestic | .16*** |
|  |  |  |  | Correlation (*r*) |  | Youngest acceptable age | -.20***/ .42*** |
|  |  |  |  |  |  | Oldest acceptable age | .48***/ -0.8*** |
|  |  |  |  | Separate logistic regression analyses (*r*) |  | Marrying someone with children | .92*** |
|  |  |  |  |  |  | Marrying someone who has been married | .87*** |
| 9 | Brumbaugh & Wood, 2013 | 17871 (M=26.4, SD=9.0)/11229 (M=27.0, SD=9.1); range? | Continuous | Correlation (*r*) | Revealed/Stated Preferences | Revealed Suggestive | -.09 to -.38/ .05 to .15 |
|  |  |  |  |  |  | Revealed Curvaceous/toned | -.22 to .15/ -.04 to .22 |
|  |  |  |  |  |  | Revealed Feminine/masculine | -.16 to .20/ -.01 to .35 |
|  |  |  |  |  |  | Revealed Confident | -.22 to .00/ -.18 to .29 |
|  |  |  |  |  |  | Revealed Conventional | .28 to .53/ .00 to .27 |
|  |  |  |  |  |  | Revealed Softhearted, sensitive | .03 to .46/ -.02 to .16 |
|  |  |  |  |  |  | Revealed Formal, classy | .21 to .42/ -.21 to 31 |
|  |  |  |  |  |  | Revealed Smiling/upbeat | -.04 to .28/ -.02 to .27 |
|  |  |  |  |  |  | Revealed Intelligent | .10 to .49/ -.09 to .19 |
|  |  |  |  |  |  | Revealed Well-groomed | .14 to .25/ -.14 to .30 |
|  |  |  |  |  |  | Revealed Trendy, stylish, urban | -.44 to -.08/ -.20 to .42 |
|  |  |  |  |  |  | Revealed Body weight | .11 to .49/ -.28 to .15 |
|  |  |  |  |  |  | Stated Suggestive | -.23 to .04/ -.06 to .24 |
|  |  |  |  |  |  | Stated Curvaceous/toned | -.08 to .13/ -.21 to .35 |
|  |  |  |  |  |  | Stated Feminine/masculine | -.02 to .19/ -.07 to .31 |
|  |  |  |  |  |  | Stated Confident | -.03 to .16/ -.10 to .17 |
|  |  |  |  |  |  | Stated Conventional | -.05 to .25/ -.23 to .06 |
|  |  |  |  |  |  | Stated Softhearted, sensitive | -.08 to .28/ -.24 to .39 |
|  |  |  |  |  |  | Stated Formal, classy | -.11 to .15/ -.09 to .09 |
|  |  |  |  |  |  | Stated Smiling/upbeat | -.16 to .18/ -.18 to .14 |
|  |  |  |  |  |  | Stated Intelligent | -.11 to .19/ -.09 to .24 |
|  |  |  |  |  |  | Stated Well-groomed | -.02 to .20/ -.12 to .19 |
|  |  |  |  |  |  | Stated Trendy, stylish, urban | -.37 to .06/ -.16 to .10 |
|  |  |  |  |  |  | Stated Body weight | -.21 to -.03/ -.11 to .21 |
| 10 | Menkin et al., 2015 | 2717/2717; 20-95 years (?, ?) | 20-91 | Censored regressions (*β*) | Importance | Sexual attraction | -.04*** |
|  |  |  |  |  |  | Interpersonal communication | .01 |
|  |  |  |  |  |  | Individual companionate characteristics | .01 |
|  |  |  | 20-39 |  |  | Sexual attraction | .18* |
|  |  |  |  |  |  | Interpersonal communication | .40*** |
|  |  |  |  |  |  | Individual companionate characteristics | .03 |
|  |  |  | 40-59 |  |  | Sexual attraction | -.26­*** |
|  |  |  |  |  |  | Interpersonal communication | -.10 |
|  |  |  |  |  |  | Individual companionate characteristics | -.02 |
|  |  |  | 60-74 |  |  | Sexual attraction | .06 |
|  |  |  |  |  |  | Interpersonal communication | .13 |
|  |  |  |  |  |  | Individual companionate characteristics | .04 |
|  |  |  | 75+ |  |  | Sexual attraction | -.04 |
|  |  |  |  |  |  | Interpersonal communication | .05 |
|  |  |  |  |  |  | Individual companionate characteristics | .10 |
| 11 | Sprecher et al., 2019 | 459/279; 18-40; (M=24.01, SD=5.27) | Continuous | Correlation (*r*) | Above average preferences | Overall selectivity composite | -.19***/-.40*** |
|  |  |  |  |  |  | Intrinsic characteristics composite | -.12**/ -.35*** |
|  |  |  |  |  |  | Social network composite | -.27***/ -.42*** |
|  |  |  |  |  |  | Resources/success composite | -.13**/ -.30*** |
|  |  |  |  |  |  | Physical appearance composite | -.14**/ -.21*** |
| 12 | Fales et al., 2016+ | 11612 (M=38, SD=12)/11203 (M=44, SD=12); 18-65 years | 18-25; 26-35; 36-45; 46-55; 56-65 | Linear regression (*β*) | Preferences (Desirability/Undesirability) | Good looking | -.24*** |
|  |  |  |  |  |  | Slender body | -.09*** |
|  |  |  |  |  |  | Steady income | -.09*** |
|  |  | 2523 (M=47, SD=15)/ 2267 (M=45, SD=15); 21-75 years | 21-25; 26-35; 36-45; 46-55; 56-65; 66-75 | Linear regression (*β*) | Importance | Makes or will make a lot of money | -.03** |
|  |  |  |  |  |  | Is physically attractive to me | -.08*** |
|  |  |  |  |  |  | Makes at least as much money as I do | -.08*** |
|  |  |  |  |  |  | Has a successful career | -.22*** |
| Note. Author names of studies using representative samples are marked with +. * *p* < .05, ** *p* < .01, *** *p* < .001. | | | | | | | |

## Target quotas

The participants were recruited following quotas respective to the Czech population according to the Czech Statistical Office^[[1]](#footnote-1)^. That is, 49% of men and 51% of women were the target quota for sex; 16% for the age group between 18 to 24 years, 32% for aged between 25 to 34 years, 34% for aged between 35 to 44, 17% for aged between 45 to 50. Quotas for the size of the residential area were set at 17% for below 999 residents, 22% between 1,000 to 4,999 residents, 18% between 5,000 to 19,999 residents, 21% between 20,000 to 99,999 residents, and 22% above 100,000 residents. Based on the highest level of education, we targeted 38% of participants with primary school, 39% with secondary school, and 23% with higher education diplomas.

| Supplementary Table S2. Comparison between the study sample and the Czech general population | | | |
| --- | --- | --- | --- |
| Variables | Study sample | General population | Comparison  *Χ^2^*(df) and *z*-tests |
| Sample | 2,280 (100%) | 10,682,029 (100%) |  |
| Sex |  |  | 10.46(1)** |
| Male | 1,040 (45.6%) | 5,234,194 (49%) |  |
| Female | 1,240 (54.4%) | 5,447,835 (51%) |  |
| Age |  |  | 15.04(3)** |
| 18-24 years | 411 (18.0%) | 1,709,125 (16%) | 2.6** |
| 25-34 years | 701 (30.7%) | 3,418,249 (32%) | -1.33 |
| 35-44 years | 732 (32.1%) | 3,631,890 (34%) | -1.92* |
| 45-50 years | 436 (19.1%) | 1,815,945 (17%) | 2.67** |
| Size of residential area |  |  | 14.66(4)** |
| 0-999 residents | 374 (16.4%) | 1,815,945 (17%) | -0.76 |
| 1,000-4,999 residents | 437 (19.2%) | 2,350,046 (22%) | -3.23** |
| 5,000-19,999 residents | 413 (18.1%) | 1,922,765 (18%) | 0.12 |
| 20,000-99,999 residents | 518 (22.7%) | 2,243,226 (21%) | 1.99* |
| above 100,000 residents | 538 (23.6%) | 2,350,046 (22%) | 1.84* |
| Education |  |  | 23.21(2)** |
| Primary school | 777 (34.1%) | 4,059,171 (38%) | -3.84** |
| Secondary school | 893 (39.2%) | 4,165,991 (39%) | 0.2 |
| Higher education | 610 (26.8%) | 2,456,867 (23%) | 4.31** |
| Note. The goodness of fit chi-square and post hoc one-proportion *z*-tests were performed to compare the observed and the expected proportions between the study sample and the general population.  * *p* < .05, ** *p* < .01. | | | |

The final resulting quotas could differ from the targets when the target number of participants fulfilled a quota, but additional participants had to be recruited to fulfill another quota which increased the final prevalence above the expected in some of the demographic strata. The final sample differed from the target quotas in age including significantly more participants aged between 18-24 and 45-50 than the general population, and significantly less between 35-44 years (Supplementary Table S1). The sample significantly differed in the size of the residential area from the general population, namely, our sample contained fewer people from the area with 1,000-4,999 residents, and more people from area with 20,000-99,999 and 100,000 and above residents. Lastly, the participants had significantly different educational levels, that is, our sample had significantly fewer participants with primary education and significantly more participants with higher education than the general population.

## All measures including those not used in the current study

The data collection took place in June 2021. The participants indicated their basic demographic data which were necessary for the representative quota (sex, age, highest level of education, and area of residence). The participants also indicated their monthly net income, their household’s monthly net income, how many people lived in the household, and how many of them were children. The participants responded whether they currently have a relationship (sexual or non-sexual) and, if not, whether they ever had a partner (sexual or non-sexual). The participants were asked about the gender of their partners/ex-partners in order to automatically give an appropriately gendered battery of questionnaires to respond to.

We asked about the current and, if single, their ex-partner’s age, highest level of education, monthly net income, and their relationship type as an open-ended question. They also indicated the length of their relationship (possible answers were: 0-3 months, 4-6 months, 7-9 months, 10-12 months, 1, 2, … 35 years, 36, and more than 36 years). Subsequently, the place where they met was indicated (the options were: school, work, party, organization, vacation, blind date, dating app, online, friends, family, co-workers, and other – specified by free text). They also rated how serious that relationship is, how satisfied they are with the relationship, how attractive they find their partner for themselves, how attractive they find their partner for others, and how attractive they find themselves for others (all questions on a 1 = ‘not at all’ to 7 = ‘very much’). They also replied about how many common children they have with this partner, and how many children their partner has apart from their common children. The same questions were answered for up to three current partners and up to three ex-partners (if currently single). In the case of the ex-partners, the participants also indicated who initiated the break-up (1 = the respondent, 2 = the partner, 3 = both of them).

Subsequently, the participants rated characteristics covering main dimensions of seven desirable (Warmth, Attractiveness, Status, Intellect, Passion, Stability, and Dominance; Csajbók & Berkics, 2017) and seven undesirable factors (Hostility, Unattractiveness, Unambitiousness, Filthiness, Arrogance, Clinginess, and Abusiveness; Csajbók & Berkics, 2022) of mating standards. Further, we included one extra factor, Depressiveness (“pessimistic and depressed”) as it was consistently found to be the most common mental health problem significantly affecting well-being and relationship satisfaction (Lim et al., 2018; Li & Johnson, 2018). All 15 factors were indicated by two characteristics that loaded on the factors in the original research (e.g., Warmth: “loving and caring”; Arrogance: “selfish and arrogant”). All items were rated on a 1 = ‘not at all’ to 7 = ‘very much’ scale. The altogether 15 items (seven desirable, eight undesirable characteristics) were rated as “To what extent do the following characteristics describe your ideal partner?”. The 15 items were rated on themselves, their ideal partners, and their current partner (or ex-partners if they were currently single). The participants had the opportunity to rate two more current (4.2% had extra-pair relationships) or ex-partners. The order of the 15 items and the targets of the ratings were all randomized.

Lastly, the participants indicated whether they were diagnosed with COVID (what type of confirmation of the diagnoses they had, i.e., symptoms or test), and how bad the course of their COVID infection was (on a 1 = ‘no symptoms’ to 6 = ‘intensive care’ scale). They also rated how badly the pandemic affected their financial, romantic, and social life, and mental, and physical health (1 = ‘not at all’ to 7 = ‘very much’). At the end, all participants had the opportunity to give feedback in text form.

| Supplementary Table S3. Correlations among self-perceived mate value and mate value indicators | | | | | |
| --- | --- | --- | --- | --- | --- |
|  | 1. | 2. | 3. | 4. | 5. |
| 1. Self-perceived mate value | − | -.02 | .01 | -.05* | -.05* |
| 2. Education | -.02 | − | .29** | <.01 | -.02 |
| 3. Personal income | .01 | .29** | − | .33** | .31** |
| 4. Age | -.05* | <.01 | .33** | − | .99** |
| 5. Age^2^ | -.05* | -.02 | .31** | .99** | − |
| * *p* < .05, ** *p* < .01. | | | | | |

| Supplementary Table S4. Correlation among mating strategy indicators | | | | | |
| --- | --- | --- | --- | --- | --- |
|  | 1. | 2. | 3. | 4. |  |
| 1. *N* children | − | .04 | .01 | .45** |  |
| 2. *N* relationships | .04 | − | .70** | -.08** |  |
| 3. *N* sex partners | .01 | .70** | − | -.11** |  |
| 4. Relationship length | .45** | -.08** | -.11** | − |  |
| * *p* < .05, ** *p* < .01. | | | | |  |

| Supplementary Table S5. Correlations between all the 15 mate preference ratings | | | | | | | | | | | | | | | |
| --- | --- | --- | --- | --- | --- | --- | --- | --- | --- | --- | --- | --- | --- | --- | --- |
|  | 1. | 2. | 3. | 4. | 5. | 6. | 7. | 8. | 9. | 10. | 11. | 12. | 13. | 14. | 15. |
| 1. loving, caring | − | .40 | .31 | .44 | .47 | .52 | .39 | -.23 | -.40 | -.27 | -.39 | -.25 | .17 | -.38 | -.31 |
| 2. attractive, physically attractive | .40 | − | .38 | .43 | .55 | .30 | .43 | -.19 | -.20 | -.19 | -.17 | -.35 | .13 | -.18 | -.22 |
| 3. good financial-, good social position | .31 | .38 | − | .43 | .33 | .32 | .40 | -.19 | -.16 | -.13 | -.13 | -.17 | .08 | -.13 | -.19 |
| 4. intelligent, educated | .44 | .43 | .43 | − | .37 | .38 | .48 | -.26 | -.27 | -.21 | -.22 | -.24 | .07 | -.25 | -.22 |
| 5. passionate, good in bed | .47 | .55 | .33 | .37 | − | .33 | .41 | -.19 | -.22 | -.20 | -.19 | -.27 | .16 | -.20 | -.25 |
| 6. calm, patient | .52 | .30 | .32 | .38 | .33 | − | .33 | -.19 | -.36 | -.24 | -.40 | -.18 | .06 | -.36 | -.32 |
| 7. purposeful, confident | .39 | .43 | .40 | .48 | .41 | .33 | − | -.29 | -.18 | -.17 | -.13 | -.22 | .09 | -.15 | -.25 |
| 8. indecisive, without ambition | -.23 | -.19 | -.19 | -.26 | -.19 | -.19 | -.29 | − | .40 | .39 | .40 | .39 | .12 | .40 | .45 |
| 9. bad, rude | -.40 | -.20 | -.16 | -.27 | -.22 | -.36 | -.18 | .40 | − | .47 | .64 | .42 | .07 | .75 | .55 |
| 10. messy, careless of hygiene | -.27 | -.19 | -.13 | -.20 | -.20 | -.24 | -.17 | .39 | .47 | − | .44 | .42 | .06 | .48 | .42 |
| 11. selfish, arrogant | -.39 | -.17 | -.13 | -.22 | -.19 | -.40 | -.13 | .40 | .64 | .44 | − | .40 | .09 | .64 | .49 |
| 12. unattractive, physically unattractive | -.25 | -.35 | -.17 | -.24 | -.27 | -.18 | -.22 | .39 | .42 | .42 | .40 | − | .05 | .40 | .40 |
| 13. emotionally dependent, demanding commitment | .17 | .13 | .08 | .07 | .16 | .06 | .09 | .12 | .07 | .06 | .09 | .05 | − | .06 | .10 |
| 14. aggressive, violent | -.38 | -.18 | -.13 | -.25 | -.20 | -.36 | -.15 | .40 | .75 | .48 | .64 | .40 | .06 | − | .54 |
| 15.pessimistic, depressed | -.31 | -.22 | -.19 | -.22 | -.25 | -.32 | -.25 | .45 | .55 | .42 | .49 | .40 | .10 | .54 | − |
| *Note.* All correlations are significant at *p* < .05. | | | | | | | | | | | | | | | |

| Supplementary Table S6. Correlation among demographic details | | | | | |
| --- | --- | --- | --- | --- | --- |
|  | 1. | 2. | 3. | 4. |  |
| 1. Relationship status | − | .14** | .22** | -.06** |  |
| 2. Household income | .14** | − | .28** | <.01 |  |
| 3. *N* people in household | .22** | .28** | − | -.22** |  |
| 4. Size of residence | -.06** | <.01 | -.22** | − |  |
| * *p* < .05, ** *p* < .01. | | | | |  |

# Supplementary references

Alterovitz, S. S. R., & Mendelsohn, G. A. (2009). Partner preferences across the life span: Online dating by older adults. *Psychology and Aging*, *24*, 513-517.

Brumbaugh, C. C., & Wood, D. (2013). Mate preferences across life and across the world. *Social Psychological and Personality Science*, *4*, 100-107.

Buunk, B. P., Dijkstra, P., Fetchenhauer, D., & Kenrick, D. T. (2002). Age and gender differences in mate selection criteria for various involvement levels. *Personal relationships*, *9*, 271-278.

Csajbók, Z., & Berkics, M. (2017). Factor, factor, on the whole, who's the best fitting of all?: Factors of mate preferences in a large sample. Personality and Individual Differences, 114, 92-102.

Csajbók, Z., & Berkics, M. (2022). Seven deadly sins of potential romantic partners: The dealbreakers of mate choice. Personality and Individual Differences, 186, 111334.

Fales, M. R., Frederick, D. A., Garcia, J. R., Gildersleeve, K. A., Haselton, M. G., & Fisher, H. E. (2016). Mating markets and bargaining hands: Mate preferences for attractiveness and resources in two national US studies. *Personality and Individual Differences*, *88*, 78-87.

Fitzpatrick, J., Sharp, E. A., & Reifman, A. (2009). Midlife singles’ willingness to date partners with heterogeneous characteristics. *Family Relations*, *58*, 121-133.

Li, P. F., & Johnson, L. N. (2018). Couples’ depression and relationship satisfaction: Examining the moderating effects of demand/withdraw communication patterns. Journal of Family Therapy, 40, S63-S85.

Lim, G. Y., Tam, W. W., Lu, Y., Ho, C. S., Zhang, M. W., & Ho, R. C. (2018). Prevalence of depression in the community from 30 countries between 1994 and 2014. *Scientific Reports, 8*, 2861–2871.

McIntosh, W. D., Locker Jr, L., Briley, K., Ryan, R., & Scott, A. J. (2011). What do older adults seek in their potential romantic partners? Evidence from online personal ads. *The International Journal of Aging and Human Development*, *72*, 67-82.

Menkin, J. A., Robles, T. F., Wiley, J. F., & Gonzaga, G. C. (2015). Online dating across the life span: Users’ relationship goals. *Psychology and Aging, 30*, 987-993.

Schwarz, S., & Hassebrauck, M. (2012). Sex and age differences in mate-selection preferences. *Human Nature*, *23*, 447-466.

South, S. J. (1991). Sociodemographic differentials in mate selection preferences. *Journal of Marriage and the Family*, *53,* 928-940.

Sprecher, S., Econie, A., & Treger, S. (2019). Mate preferences in emerging adulthood and beyond: Age variations in mate preferences and beliefs about change in mate preferences. *Journal of Social and Personal Relationships*, *36*, 3139-3158.

Tadinac, M., & Hromatko, I. (2007). Own mate value and relative importance of a potential mate's qualities. *Studia Psychologica*, *49*, 251-264.

Wood, D., & Brumbaugh, C. C. (2009). Using revealed mate preferences to evaluate market force and differential preference explanations for mate selection. *Journal of Personality and Social Psychology*, *96*, 1226-1244.

1. <https://www.czso.cz/> [↑](#footnote-ref-1)
